# The mechanism of oxymatrine on atopic dermatitis in mice based on SOCS1/JAK-STAT3 pathway

**DOI:** 10.3389/fphar.2022.1091090

**Published:** 2023-01-10

**Authors:** Xianwei Han, Tianming Ma, Qiang Wang, Chunlin Jin, Yusheng Han, Guijun Liu, Hao Li

**Affiliations:** ^1^ Department of Dermatology, The Seventh People’s Hospital of Shenyang, Shenyang, China; ^2^ The Second Hospital of Heilongjiang University of Chinese Medicine, Harbin, China; ^3^ Heilongjiang University of Chinese Medicine, Harbin, China; ^4^ Shenzhen University General Hospital, Shenzhen, China

**Keywords:** oxymatrine, SOCS1, janus kinase, JAK-STAT3, atopic dermatitis

## Abstract

Based on the suppressor of cytokine signaling 1 (SOCS1)/Janus kinase (JAK)/signal transducer and activator of transcription 3 (STAT3) pathway, the mechanism of oxymatrine in the treatment of atopic dermatitis (AD) was preliminarily explored in this study. C57BL/6 mice were induced to establish AD model by smearing carbotriol (MC903) on their back. The AD mice were randomly divided into model group, oxymatrine groups with three dosages (25, 50 and 100 mg/kg), (*n* = 10). Oxymatrine groups were intragastric administered once daily for 14 days. The same volume of saline was given in the normal control group and model group once daily for 14 days. Subsequently, HE staining was used to observe the pathological changes of skin tissue, ELISA was used to detect the levels of serum inflammatory factors including interleukin-4, 6 and 17 (IL-4, IL-6, and IL-17), tumor necrosis factor-α (TNF-α) and immunoglobulin E (IgE). Immunohistochemistry was used to detect the expression of suppressor of cytokine signaling 1 and CD3 in skin tissue, and Western blotting was used to detect the proteins in suppressor of cytokine signaling 1/JAK-STAT3 pathway. Compared with the normal control group, the pathological damage of mice in the model group, such as skin hyperplasia, edema, congestion and inflammatory infiltration, aggravated increased significantly. And the expression of serum inflammatory factors, CD3 positive expression and JAK-STAT3 pathway protein in the model group were increased (*p* < .05), and the expression of suppressor of cytokine signaling 1 protein (*p* < .05) was decreased. Compared with the model group, the above pathological damage of the mice was reduced, and the serum inflammatory factors, JAK-STAT3 pathway protein, and CD3 positive expression were decreased as a dose-dependant manner (*p* < .05), and the expression of suppressor of cytokine signaling 1 protein was increased as a dose-dependent manner (*p* < .05). Oxymatrine can improve the skin inflammation symptoms of AD mice by up regulating the expression of suppressor of cytokine signaling 1, inhibiting the activation of JAK-STAT3 pathway and blocking the activation of T lymphocytes.

## 1 Introduction

Atopic dermatitis (AD) is a chronic inflammatory skin disease that was related to autoimmunity (Kim et al., 2019). AD could cause some complications, such as metabolic disorders, cardiovascular diseases, and emotional disorders, which seriously affect the quality of life of patients (Robert et al., 2018; Tsakok et al., 2019). At present, the etiology and pathogenesis of AD are still unclear. It is generally believed that it was caused by the interaction of multiple complex factors such as genetics, immune disorders, epidermal gene mutations, and environment and microorganisms, and is highly correlated with the helper T cell 2 (Th2) and helper T cell 22 (Th22) cell response drive (Liang et al., 2021).

Clinical treatment of AD is to relieve or eliminate clinical symptoms, eliminate inducing factors, reduce and prevent recurrence, and improve the quality of life of patients (Boothe et al., 2017; Tiago et al., 2019). So, external drugs such as glucocorticoids, phosphodiesterase 4 (PDE-4) inhibitors, and systemic drugs such as oral antihistamines, immunosuppressive agents, biological agents, Janus kinase inhibitors are the main clinical drugs in the treatment of AD (Liu et al., 2022).

Due to the chronic and recurrent characteristics of AD, patients need to receive long-term treatment, looking for good efficacy and less adverse reactions of drugs has been of concern. Oxymatrine (OMT), a common matrine-type alkaloid with quinolizidine structure, was the main active ingredient in the herb of *Sophora flavescens* Alt., *Sophora tonkinensis* Gagnep and *Sophora alopecuroides* L. OMT and the pharmaceutical preparation with OMT have been widely used in the clinical treatment of a variety of diseases, and showed a certain therapeutic effect (Lan et al., 2020; Lan et al., 2021). Clinically, OMT injection has been used in the treatment of chronic hepatitis B and C, herpes zoster, liver fibrosis and other diseases (Song et al., 2016). The therapeutic effect is obvious and the related symptoms are effectively alleviated. OMT has a variety of pharmacological effects, such as anti-infection, anti-virus, sedative and hypnotic, antipyretic and analgesic, and immune regulation (Wu and Gao, 2015; Clarissa et al., 2019; Wang et al., 2021). It has been reported that OMT has a certain therapeutic effect on atopic dermatitis (AD) model mice, and its mechanism is related to inhibiting the infiltration of mast cells in the dermis and reducing the expression of Th2 cytokines (Sun et al., 2021).

Suppressor of cytokine signaling 1 (SOCS1) can effectively avoid excessive immune response and prevent the development of chronic inflammatory diseases by negatively regulating Janus kinase (JAK)/signal transducer and activator of transcription 3 (STAT3) (Ho et al., 2020). Therefore, this study established an AD model in mice to explore the therapeutic mechanism of oxymatrine on AD from the perspective of SOCS1/JAK-STAT3 pathway, in order to find specific and effective drugs for the treatment of AD.

## 2 Materials and methods

### 2.1 Reagents

Oxymatrine (Y30S6-Y17043, HPLC grade, purity ≥ 98%) was purchased from Shanghai Yuanye Bio-Technology Co., Ltd. Interleukin-4 (IL-4) ELISA kit (EK0405), interleukin-6 (IL-6) ELISA kit (EK0411), interleukin-17 (IL-17) ELISA kit (EK0431) and tumor necrosis factor-*α* (TNF-α) ELISA kit (EK0527) were purchased from Wuhan Boster Biological Engineering Co., Ltd. Immunoglobulin E (IgE) ELISA kit (bsk12030) was purchased from Beijing Bioss Biotechnology Co., Ltd. Rabbit anti-mouse antibodies such as SOCS1 (ab280886), T lymphocyte surface marker CD3 (ab5690), JAK1 (ab125051), STAT3 (ab68153), and phosphorylated JAK1 (p-JAK1) (ab1380056) were purchased from the Abcam Co., Ltd.

### 2.2 Animals and treatment

50 healthy male C57BL/6 mice, the weight range of which was 18–22 g, were purchased from Beijing Vital River Laboratory Animal Technology Co., Ltd. with the certificate number of SCXK (Jing) 2021-006. The AD model was established with applying 20 μl carbotriol (MC903) solution (2 nmol/L) to the dorsum for 10 days according to the method with minor modification (Chang et al., 2020). AD model was successful when the ear skin of mice has the phenomena of scratches, erosion, erythema bleeding, and scab. 40 AD mice were randomly divided into model group, oxymatrine group (25 mg/kg, OMT-L), oxymatrine group (50 mg/kg, OMT-M), and oxymatrine group (100 mg/kg, OMT-H) (*n* = 10). Oxymatrine groups were intragastric administered once daily for 14 consecutive days, and the same volume of saline was given in the normal control group and model group once daily for 14 consecutive days. All the mice were anesthetized 2 h after administration on the 14th day. Serum was obtained after the blood samples were collected and centrifugated at 3000 rpm for 15 min at 4°C.

### 2.3 Detection of TNF-α, IgE, IL-4, IL-6, and IL-17 by ELISA

After the end of the last administration, blood was taken from the abdominal aorta of the mice after anesthesia to obtain the serum, and the contents of TNF-α, IgE, IL-4, IL-6, and IL-17 in the serum were detected according to the instructions of the ELISA kit.

### 2.4 Histopathological observation of skin

The dorsum tissue of mice was cut and separated. Some of the samples were quickly stored in liquid nitrogen for standby, and the other part was fixed in 4% paraformaldehyde for 24 h. The sections with a size of 5 μm were made after conventional paraffin embedding, and the histopathological changes of skin in each group were observed under microscope after HE staining.

### 2.5 Detection of SOCS1 and T lymphocyte surface marker CD3 positive expression by immunohistochemical

The paraffin sections of skin tissue were dewaxed and antigen repaired, then the SOCS1 and CD3 rabbit anti mouse antibodies with the ratio of 1:200 were dripped and incubated overnight at 4°C. After the biotin labeled IgG antibody was dripped and incubated at room temperature for 2 h, DAB staining and hematoxylin re-staining were performed, and then observed and photographed under the microscope. Image-Pro Plus 6.0 software was used to analyze the average optical density value of positive staining under each field of vision.

### 2.6 Detection of SOCS1/JAK-STAT3 pathway protein by western blotting

Skin tissue was weighted and the protein of the tissue was extracted after grinding with liquid nitrogen. BCA method was used to detect the protein concentration. 50 μg of protein was subjected to electrophoresis and membrane transfer reaction, and the primary antibodies of SOCS1, JAK1, p-JAK1, STAT3, p-STAT3 with the ratio of 1:1,000 and the antibody of β-actin with the ratio of 1:2,000 were dropped for incubating overnight at 4°C. HRP goat anti rabbit secondary antibody with the ratio of 1:3,000 was dropped and incubated at room temperature for 2 h. After enhanced chemiluminescence color exposure, the relative expression of the bands was analyzed by ImageJ software.

### 2.7 Statistical methods

SPSS 24.0 software was used for statistical analysis. The measurement data conforming to the normal distribution was expressed by mean ± standard deviation (*x ± s*). Single factor analysis of variance was used for comparison between groups, and *t*-test was used for pairwise comparison between groups.

## 3 Results

### 3.1 Effect of OMT on histopathological damage of skin tissue

**FIGURE 1:**
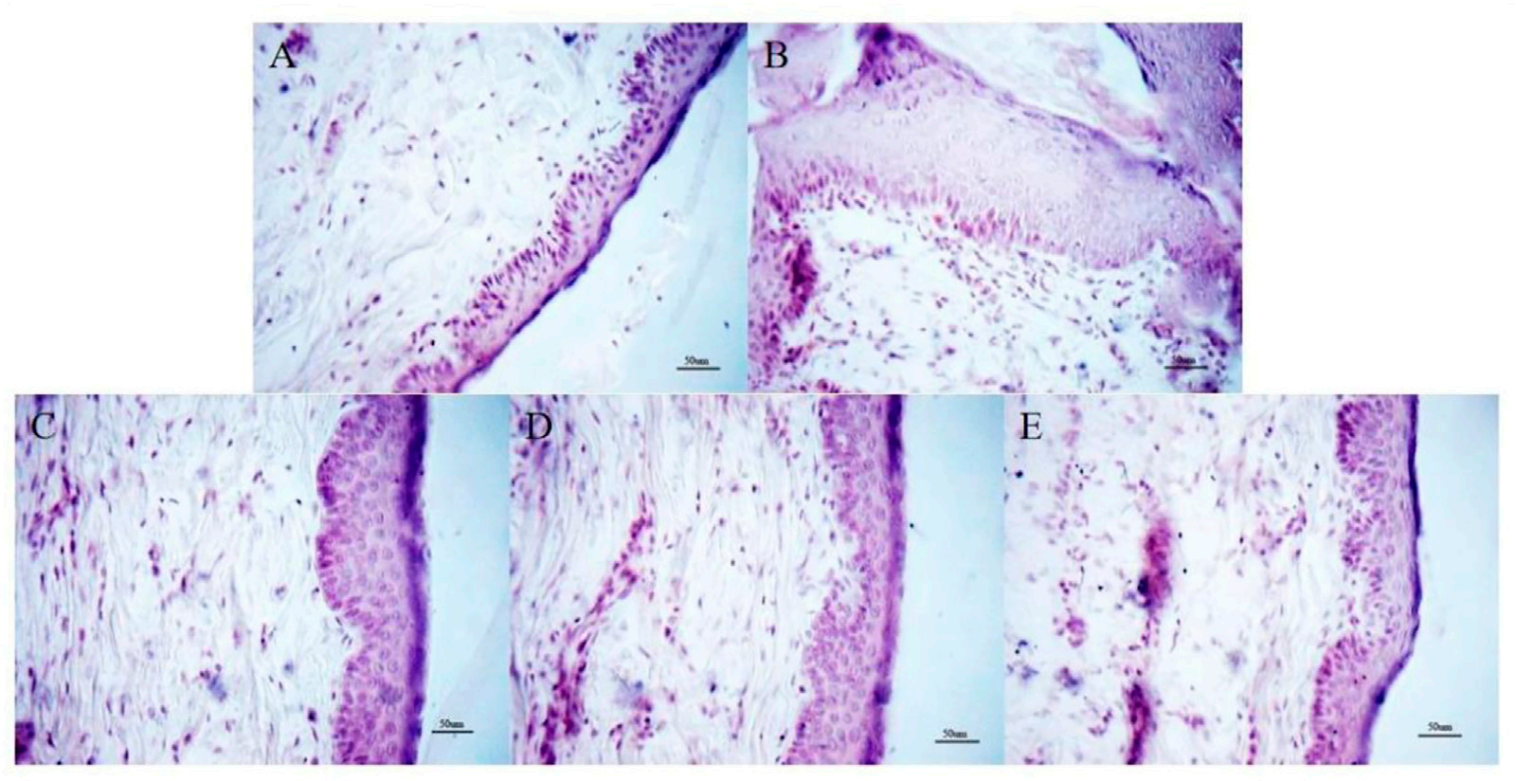
Histopathological changes of skin tissue (HE staining, × 200). **(A)** Control group; **(B)** Model group. **(C)** OMT-H group. **(D)** OMT-M group. **(E)** OMT-L group.

The skin texture of mice in the control group was clear and normal without pathological symptoms. In the model group, the pathological symptoms such as hyperplasia of epidermis, thickening of acanthocyte layer, tissue edema and congestion, telangiectasia, and infiltration of inflammatory cells were obvious; The above pathological damage of OMT-H, OMT-M and OMT-L groups were significantly reduced compared with that of model group (Figure 1).

### 3.2 Effect of OMT on TNF-α, IgE, IL-4, IL-6 and IL-17 levels in serum

**FIGURE 2:**
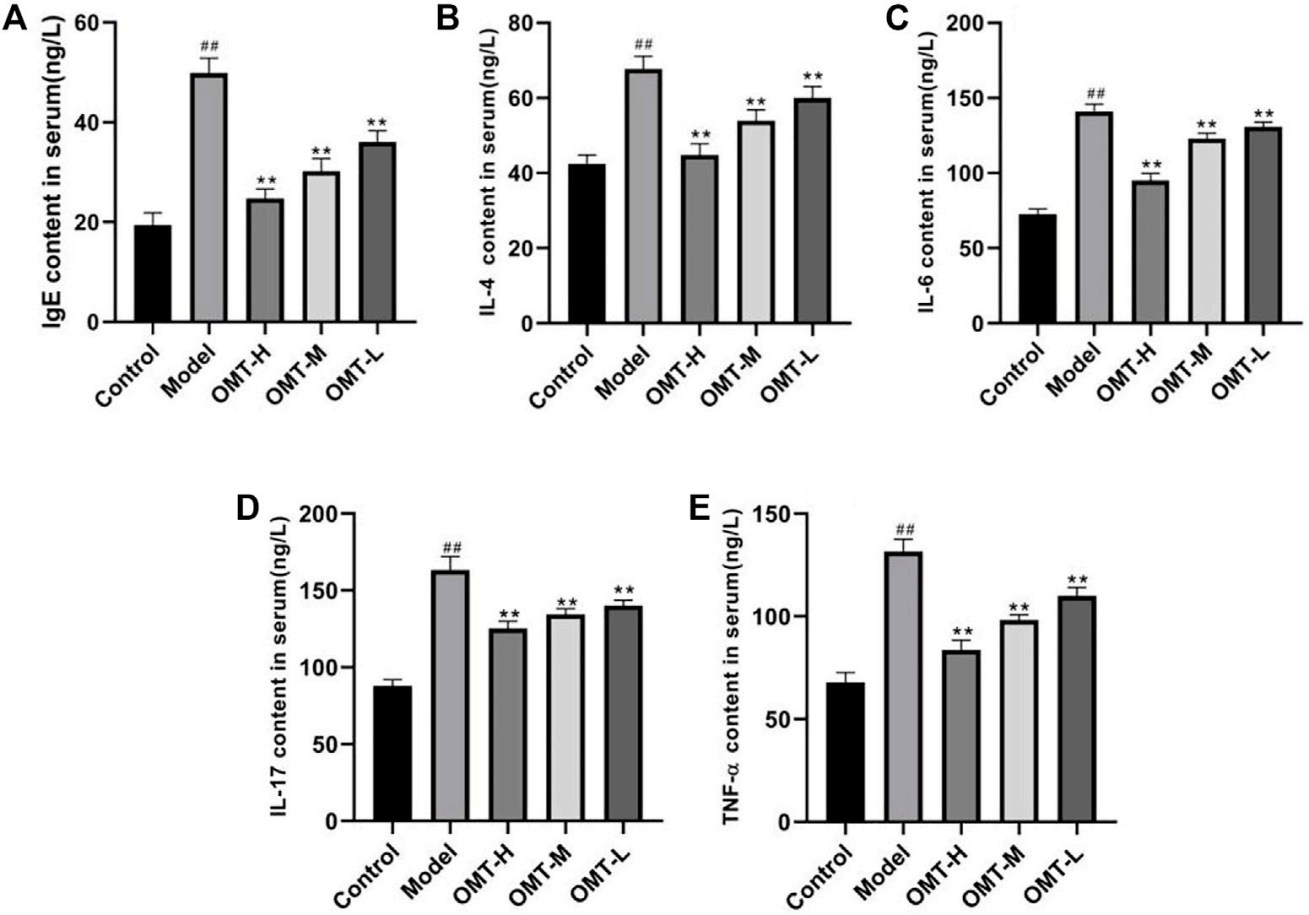
Comparison of **(A)** IgE, **(B)** IL-4, **(C)** IL-6, **(D)** IL-17 and **(E)** TNF-α levels in serum. (^##^, *p* < .01, vs. Control group; **, *p* < .01, vs. model group).

Compared with the normal control group, the TNF-α, IgE, IL-4, IL-6 and IL-17 levels in serum of the mice in model group increased (*p* < .05). Compared with the model group, the TNF-α, IgE, IL-4, IL-6, and IL-17 levels in serum of the mice in OMT-H, OMT-M and OMT-L groups decreased as a dose-dependent manner (*p* < .05) (Figure 2).

### 3.3 Effect of OMT on T lymphocyte infiltration in skin tissue

**FIGURE 3:**
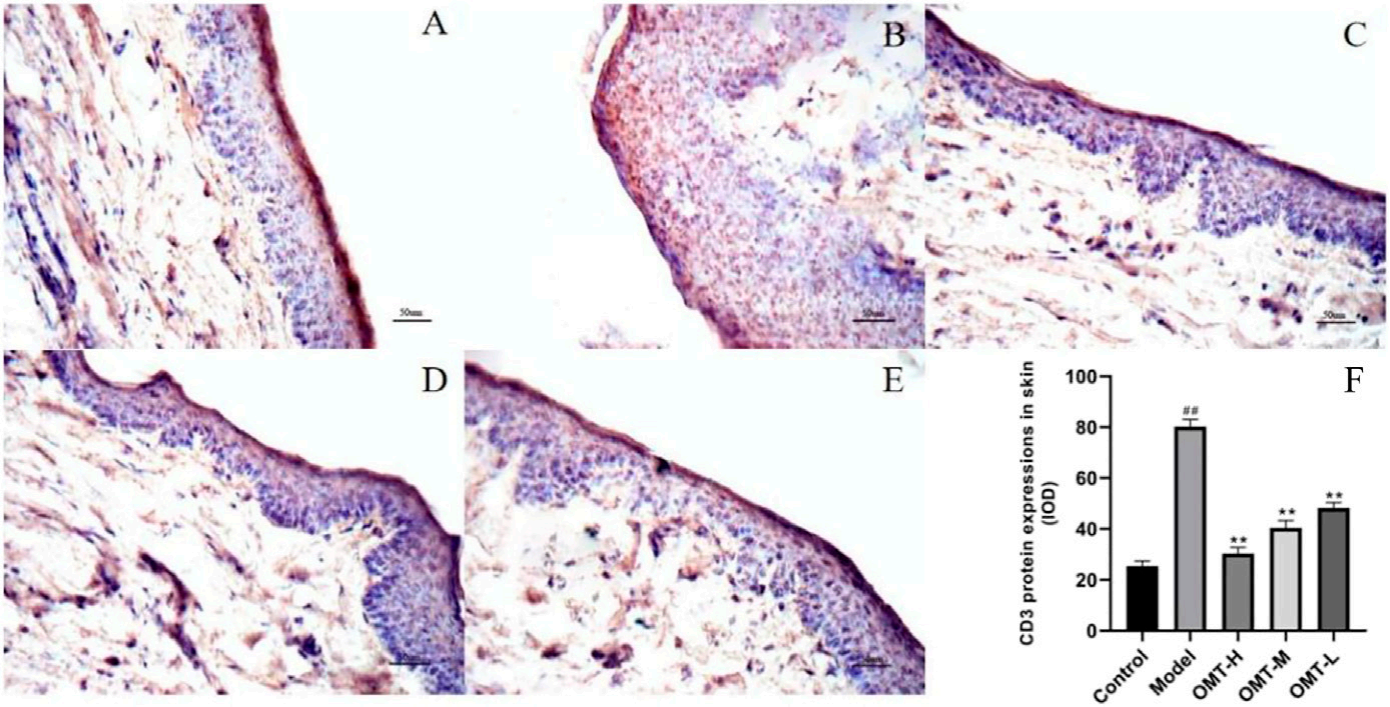
Positive expression of CD3 in skin tissue (immunohistochemical staining, × 200). **(A)** Normal control group; **(B)** Model group. **(C)** OMT-H group. **(D)** OMT-M group. **(E)** OMT-L group. **(F)** Statistical data of positive expression of CD3 in each group. (^##^, *p* < .01, vs. Control group; **, *p* < .01, vs. model group).

The positive expression of CD3 can reflect the infiltration of T lymphocytes. Compared with the normal control group, the positive expression of CD3 in the skin tissue of the model group increased (*p* < .05). Compared with the model group, the positive expression of CD3 in the skin tissue of mice in the OMT-H, OMT-M, and OMT-L groups decreased as a dose-dependent manner (*p* < .05) (Figure 3).

### 3.4 Effect of OMT on the expression of SOCS1 in skin tissue

**FIGURE 4:**
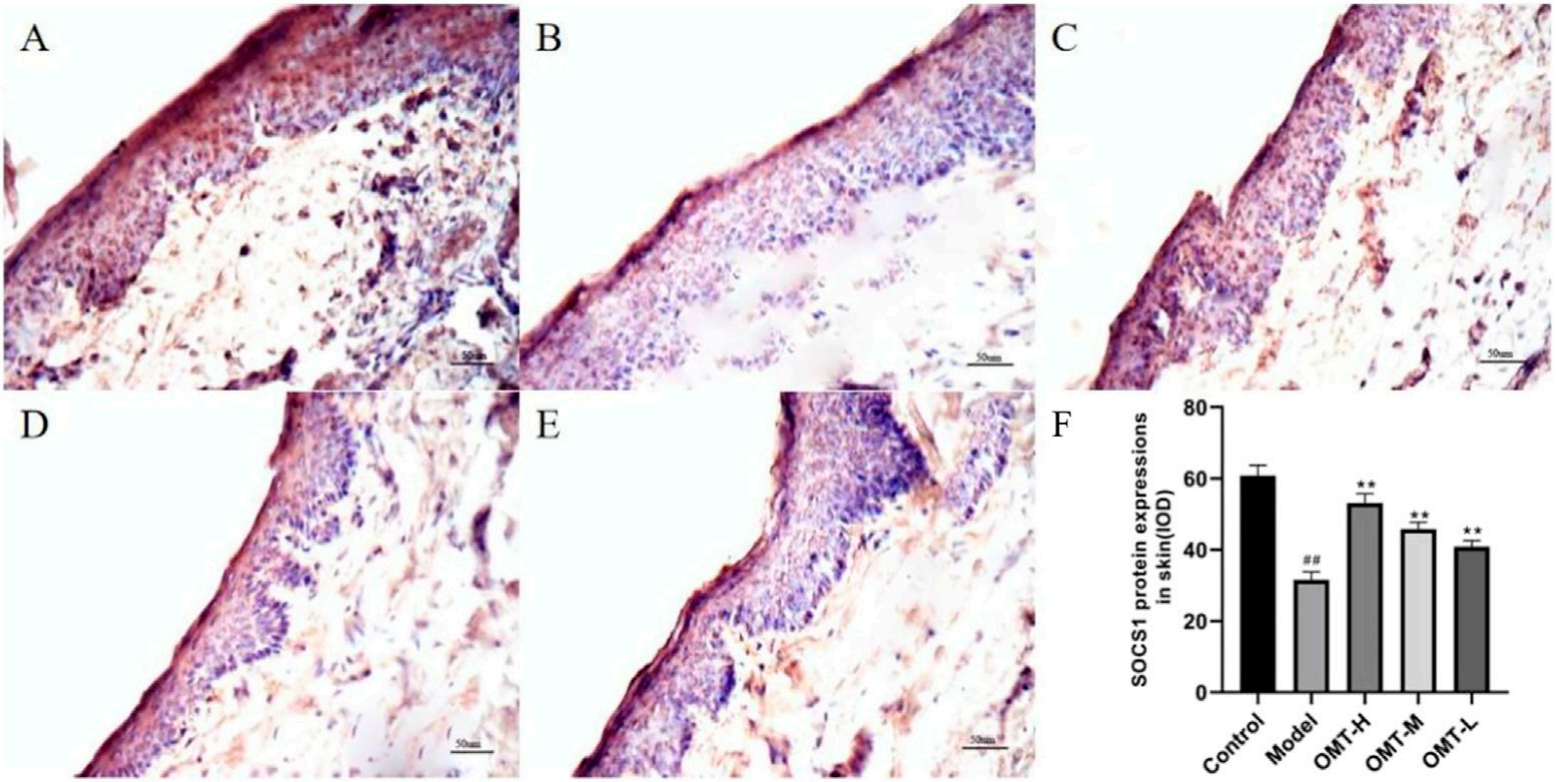
Positive expression of SOCS1 in skin tissue (immunohistochemical staining, × 200). **(A)** Normal control group; **(B)** Model group. **(C)** OMT-H group. **(D)** OMT-M group. **(E)** OMT-L group. **(F)** Statistical data of positive expression of SOCS1 in each group. (^##^, *p* < .01, vs. Control group; **, *p* < .01, vs. model group).

**FIGURE 5:**
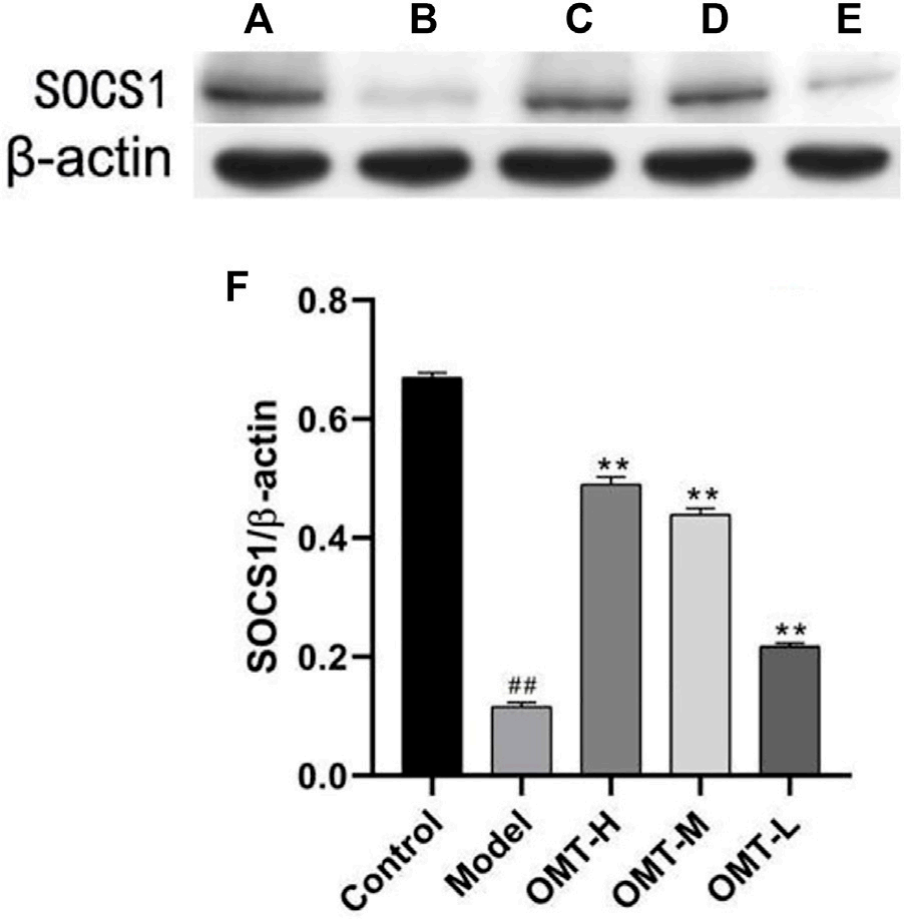
Expression of SOCS1 protein in skin tissue (β-actin image for control were the same in Figure 5 and Figure 6). **(A)** Normal control group; **(B)** Model group. **(C)** OMT-H group. **(D)** OMT-M group. **(E)** OMT-L group. **(F)** Statistical data of expression of SOCS1 protein in each group. (^##^, *p* < .01, vs. Control group; **, *p* < .01, vs. model group).

The positive expression of SOCS1 was brownish yellow granules. Compared with the normal control group, the brownish yellow particles in the skin tissue of the model group decreased, and the positive expression of SOCS1 and protein expression decreased (*p* < .05); Compared with the model group, the positive expression of SOCS1 and protein expression in skin tissue of mice in the OMT-H, OMT-M, and OMT-L groups increased as a dose-dependent manner (*p* < .05) (Figures 4, 5).

### 3.5 Effect of OMT on expression of JAK-STAT3 pathway protein in ear tissue

**FIGURE 6:**
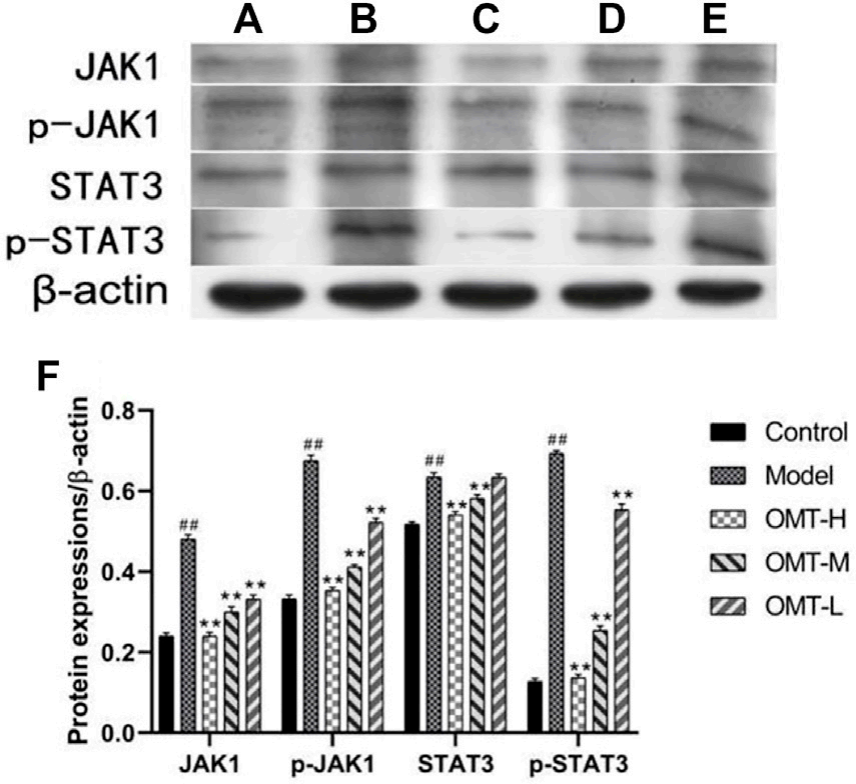
Expression of JAK1, p-JAK1, STAT3 and p-STAT3 protein in skin tissue (β-actin image for control were the same in Figure 5 and Figure 6). **(A)** Normal control group; **(B)** Model group. **(C)** OMT-H group. **(D)** OMT-M group. **(E)** OMT-L group. **(F)** Statistical data of expression of JAK1, p-JAK1, STAT3 and p-STAT3 protein in each group. (^##^, *p* < .01, vs. Control group; **, *p* < .01, vs. model group).

Compared with the normal control group, the expression of p-JAK1, JAK1, p-STAT3, and STAT3 protein in the skin tissue of mice in the model group increased (*p* < .05). Compared with the model group, the expression of p-JAK1, JAK1, p-STAT3 protein in skin tissue of mice in OMT-H and OMT-L groups decreased as a dose-dependent manner (*p* < .05), and the expression of STAT3 protein in skin tissue of mice in OMT-H and OMT-M groups decreased (*p* < .05). The expression of STAT3 protein in OMT-L group was not statistically significant (Figure 6).

## 4 Discussion

The cure rate of AD is low, and the incidence rate of AD is high in children. At present, studies have found that the activation of inflammatory reaction mediated by the interaction of activated T lymphocytes, eosinophils and mast cells is the key mechanisms of AD skin damage (Demeyer et al., 2019). After allergen contacts with skin to form complex, it can induce antigen presenting cells in skin to activate and present allergen to T lymphocytes, stimulate T lymphocytes to activate and transform into Th2 cell subsets and release IL-4, IL-17 and other inflammatory factors to aggravate skin damage (Wu et al., 2021). In addition, the expansion of T lymphocytes to Th2 cell subsets will also promote the conversion of B cells and recognize IgE antibodies of specific environmental allergens, thus causing type I hypersensitivity. The levels of IgE, TNF-α, IL-4, IL-7 and other inflammatory factors in the serum of AD patients are abnormal and higher than that of normal people. The higher the level of IgE, TNF-α, IL-4 and IL-7, the more serious the condition of AD patients (Pei et al., 2020). Inhibiting skin inflammatory damage and regulating immune response are currently widely accepted therapeutic strategies for AD (Hu and Zhang, 2019). In this study, it was found that at the same time as the levels of IgE, TNF-α, IL-4 and IL-7 in serum of mice in the model group increased, the positive expression of CD3, the surface marker of T lymphocytes in skin tissue, was also increased, and the pathological manifestations such as skin epidermal hyperplasia, congestion, edema, and inflammatory cell infiltration were significantly aggravated, suggesting that mice had T lymphocyte activation and inflammatory damage, indicating that the model was established successfully.

OMT is one of the active ingredients in *S. flavescens* Alt., *S. tonkinensis* and *S. alopecuroides* L. Modern clinical studies have found that it has a variety of pharmacological effects such as anti-virus, anti-inflammatory, anti-fibrosis, immune regulation, etc. Studies have shown that OMT can inhibit the contact dermatitis and lymphocyte proliferation induced by dinitrofluorobenzene in mice, improve the symptoms of skin lesions, and significantly reduce the inflammatory reaction (Wu et al., 2005; Wu et al., 2006), indicating that OMT may be a potential drug for the treatment of AD.

In this study, after the intervention treatment with different doses of OMT in mice, it was found that the serum inflammatory factors IgE, TNF-α, IL-4 and IL-7 decreased, the positive expression of CD3 in skin tissue decreased, and the pathological damage such as skin epidermal hyperplasia and inflammatory cell infiltration also eased significantly, indicating that OMT has potential application value in the treatment of AD.

SOCS1/JAK-STAT3 pathway is one of the important pathways that mediate the body’s immune inflammatory response. A large number of studies have found that the kinase inhibition region sequence of SOCS1 can inhibit the activation of STAT3 by JAK2 kinase, prevent the production of IL-23 and IL-17, and reduce the inflammatory damage of tissues (Oh et al., 2019). In addition, immunogens and allergens stimulate the body to secrete inflammatory factors such as IL-6 or IL-23, stimulate JAK-STAT pathway, and generate activated p-STAT3 to promote the expression of Th17 transcription factor-ROR*γ*T in T-lymphoid subsets, which can promote the expression of inflammatory factors such as IL-17, aggravate the occurrence of immune diseases (Dhanushkodi et al., 2020), inhibit the activation of JAK-STAT3 pathway, and improve the symptoms of AD lesions (Li et al., 2021). The generation of SOCS1 can combine with JAKs to inhibit the over activation of JAK-STAT signal cascade reaction generated by IL-4, IL-6 and other stimuli, thus alleviating tissue damage (Luan et al., 2020). This study found that in the AD model group, the expression of SOCS1 in the ear skin tissue was decreased, the expression of its downstream JAK-STAT3 pathway and T lymphoid activation related promoters IL-6 and IL-17 were increased, and the AD-like pathological damage of skin was aggravated, suggesting that the decreased expression of SOCS1 and the continued activation of JAK-STAT3 might be one of the reasons for aggravation of skin inflammatory damage mediated the activation of T lymphocytes in AD mice. The expression of SOCS1 in the ear skin of mice in the OMT-H, OMT-M and OMT-L groups increased, and the expression of JAK-STAT3 pathway and T lymphoid activation related promoters decreased, suggesting that OMT could improve AD skin inflammation symptoms, which may be related to the up regulation of SOCS1 expression, inhibition of JAK-STAT3 pathway and T lymphoid activation.

To sum up, OMT can up regulate the expression of SOCS1, inhibit the activation of JAK-STAT3 pathway, block the activation of T lymphocytes, improve the skin inflammatory symptoms of AD mice. This study provided some theoretical basis in the field of the treatment on AD by OMT.

## Data Availability

The original contributions presented in the study are included in the article/Supplementary Material, further inquiries can be directed to the corresponding authors.
